# Leprosy Reactions in Patients Coinfected with HIV: Clinical Aspects and Outcomes in Two Comparative Cohorts in the Amazon Region, Brazil

**DOI:** 10.1371/journal.pntd.0003818

**Published:** 2015-06-01

**Authors:** Carla Andréa Avelar Pires, Fernando Octávio Machado Jucá Neto, Nahima Castelo de Albuquerque, Geraldo Mariano Moraes Macedo, Keila de Nazaré Madureira Batista, Marília Brasil Xavier

**Affiliations:** 1 Department of Dermatology, Division of Tropical Skin Diseases, Section of Leprosy, Universidade Federal do Pará, Belém, Pará, Brazil; 2 Department of Dermatology, Division of Tropical Skin Diseases, Section of Leprosy, Universidade do Estado do Pará, Belém, Pará, Brazil; 3 Department of Infectious Diseases, Division of Tropical Diseases, Section of Leprosy, Universidade Federal do Pará, Belém, Pará, Brazil; 4 Department of Infectious Diseases, Division of Tropical Diseases, Section of Epidemiological Vigilance, Universidade Federal do Pará, Belém, Pará, Brazil; Fondation Raoul Follereau, FRANCE

## Abstract

**Background:**

Leprosy, caused by *Mycobacterium leprae*, can lead to scarring and deformities. Human immunodeficiency virus (HIV), a lymphotropic virus with high rates of replication, leads to cell death in various stages of infection. These diseases have major social and quality of life costs, and although the relevance of their comorbidity is recognized, several aspects are still not fully understood.

**Methodology/Principal Findings:**

Two cohorts of patients with leprosy in an endemic region of the Amazon were observed. We compared 40 patients with leprosy and HIV (Group 1) and 107 leprosy patients with no comorbidity (Group 2) for a minimum of 2 years. Group 1 predominantly experienced the paucibacillary classification, accounting for 70% of cases, whereas Group 2 primarily experienced the multibacillary classification (80.4% of cases). There was no significant difference in the prevalence of leprosy reactions among the two groups (37.5% for Group 1 vs. 56.1% for Group 2), and the most frequent reaction was Type 1. The appearance of Group 1 patients’ reversal reaction skin lesions was consistent with each clinical form: typically erythematous and infiltrated, with similar progression as those patients without HIV, which responded to prednisone. Patients in both groups primarily experienced a single episode (73.3% in Group 1 and 75% in Group 2), and Group 1 had shorter reaction periods (≤3 months; 93.3%), moderate severity (80%), with 93.3% of the patients in the state of acquired immune deficiency syndrome, and 46.7% presenting the reaction at the time of the immune reconstitution inflammatory syndrome.

**Conclusions/Significance:**

This study used a large sample and makes a significant contribution to the clinical outcomes of patients in the reactive state with comorbid HIV and leprosy. The data indicate that these diseases, although concurrent, have independent courses.

## Author Summary

Leprosy and HIV infections, separately, are serious modern public health problems. Many studies have been conducted on these diseases, but knowledge gaps remain. This article provides the first account of important clinical information on a significant sample of patients with leprosy, as well as patients with both leprosy and HIV, who were followed over a period of 24 months. We compared the clinical outcome of both groups, observed the occurrence of reactional episodes, and examined the characteristics of these episodes. The sample consisted of 40 co-infected patients (Group 1) and 107 patients with leprosy only (Group 2). Group 1 was characterized by high levels of paucibacillary leprosy cases (70%) and the borderline tuberculoid clinical form (45%), while Group 2 predominantly exhibited multibacillary leprosy (86%) and the borderline clinical form (40.2%). The Type I reaction was present in 13 and 34 patients of Groups 1 and 2, respectively. The Amazon region, where the study was conducted, is an endemic region for both diseases, which can be useful for conducting studies such as these owing to the generalizability of the results. This study seeks to contribute to the knowledge of the natural history of HIV and leprosy comorbidity.

## Introduction

Leprosy, a chronic infectious disease caused by *Mycobacterium leprae*, can cause scars and deformities, especially if not treated quickly [1]. Brazil is currently responsible for approximately 92% of leprosy cases in the Americas, and is ranked second, behind India, in the number of global cases [2]. Despite the number of detected leprosy cases in the country remaining stable, the North, Midwest, and Northeast regions are the most heavily affected, in proportion to the population [3].

Human immunodeficiency virus (HIV) is a lymphotropic virus belonging to the *Retroviridae* family, which maintains high rates of viral replication, causing cell death in all infection stages [4]. Early diagnosis and clinical management of HIV and its complications are often complex. With the advent of antiretroviral therapy, there has been great improvement in the prognosis and quality of life of people living with HIV [5]. However, due to the increased number of people living with this virus, HIV prevalence continues to increase even in leprosy-endemic countries, which increases the risk of comorbidity [6].

Since the first report of a comorbid infection in a patient with HIV and *M*. *leprae*, several questions have been raised regarding the consequences of their interaction, especially considering the direct involvement of T-helper CD4+ lymphocytes in the pathogenesis of both diseases. Early records of this co-infection theory reported that patients developed serious forms of infection due to their immune suppression caused by HIV; however, many studies have shown no or limited alterations in the course of patients with a leprosy and HIV comorbidity [7].

Regarding the interaction conditions of the two infections, a decrease in frequency and intensity was expected, since these are both immune-mediated phenomena. However, research and reports on the subject have shown the continued occurrence of leprosy, including recent data showing that co-infected patients had stronger reactions to the diagnosis (31.5% vs. 18.8%) compared with the group without HIV [8]. However, during the vigilance period of reaction rates in groups, both were similar (59.3% vs. 53.1%). Neural damage was also expected since HIV patients are also at risk of developing lesions in their generalized peripheral nerves, including mono-neuropathy and peripheral neuritis multiplex through both HIV infection and the treatment itself [9].

The introduction of antiretroviral therapy has created, in itself, a new clinical syndrome, which is called reconstitution inflammatory syndrome or immune reconstitution inflammatory syndrome. This syndrome affects HIV-positive patients who are in an advanced stage of the disease (CD4 <200/ml). In these patients, the clinical signs of inflammation associated with opportunistic infections are usually an immune response during the transition process, in which the viral load decreases and T-helper CD4+ cell count increases by more than 20% [7,10–14].

Several authors describe the leprosy reaction at the start of the clinical manifestation of leprosy, as part of a demonstration of immune reconstitution inflammatory syndrome [7,15–19]. From 2002 to 2009, 21 cases of reversal reactions occurred as a manifestation of immune reconstitution inflammatory syndrome; of these 21 cases, 13 were diagnosed in Brazil [6]. Altogether, these cases have been reported primarily in areas where antiretroviral therapy is no longer available: 70% in South America (with 58% being from Brazil) and 20% in India.

Lockwood and Lambert proposed a definition of a leprosy immune reconstitution inflammatory syndrome event in 2010 [9] to facilitate its correct identification. This can even be recognized by the following characteristics: (1) clinical symptoms of leprosy and/or leprosy reaction starting within 6 months of antiretroviral therapy; (2) advanced HIV infection; (3) CD4+ cell counts <200 cells/mm^3^ before initiating highly active antiretroviral therapy; and (4) increased CD4+ cells in the peripheral blood after highly active antiretroviral therapy [6].

By observing the magnitude of the two diseases in northern Brazil, particularly in the state of Pará, research on leprosy and HIV comorbidity allows the monitoring of the clinical outcomes of patients, including observations of reaction aspects of the phenomena.

## Methods

Using a comparative study of clinical features, we analyzed two cohorts of patients with leprosy. Group 1 was made up of 40 patients with leprosy and HIV, while Group 2 consisted of 107 leprosy patients, all registered at the clinic of infectious dermatology, in the Núcleo de Medicina Tropical da Universidade Federal do Pará. The first cohort was examined from 2007 until May of 2013. Patients were followed from early diagnosis and the start of their specific multidrug leprosy therapy for a minimum of 2 years. Most participants were adults, and had provided informed consent in agreeing to participate in the study. For those participants who were under 18 years old, the informed consent was provided by their parents.

Patients allocated into Group 1 had previously been diagnosed as HIV-positive by serological selection using enzyme-linked immunosorbent assays (ELISA) and two confirmatory tests (western blot), had either been treated with highly active antiretroviral therapy or had not been treated, and showed signs and symptoms of leprosy as outlined by the Ministry of Health. Their diagnosis of leprosy was also supplemented by additional tests (acid-fast bacilli and histopathology). The second clinical cohort, Group 2, included patients diagnosed with leprosy according to the Ministry of Health signs and symptoms [20], and their diagnosis was supplemented by the aforementioned additional tests, presenting negative results obtained through immunochromatography (Abbott).

The criteria adapted by Andrade, Lehman, and Schureuder [21], of the International Federation of Anti-Leprosy Associations (ILEP) manual, were used to classify the intensity and frequency of reactional states, dividing reactive episodes by their intensity into mild, moderate, and severe. Using this classification, a mild reversal reaction was classified as only affecting the skin, with increased erythema and infiltration of pre-existing lesions, with no ulceration, effect on the nerve trunk, or new lesions. Type 2 reactions are considered mild when they produce fewer than 10 nodes per body segment, with the absence of systemic symptoms. Type 1 reactions are called moderate when pre-existing injuries are more erythematous, swollen, and painful, and new lesions arise. In these cases, no systemic or nerve involvement occurs. In a Type 2 reaction, systemic involvement and fever are both moderate, there is no neuritis, and 10–20 nodes are presented per affected body segment, with more than one segment affected. Severe reversal reactions, on the other hand, affect nerves, exacerbate existing lesions, cause new lesions, and can affect the eyes. Type 2 reactions have more than 20 nodes, multiple affected body segments, nodules that are spontaneously painful, and an intense systemic involvement with high fever. Periodicity was classified as subintrant or recurrent. The former was characterized by a good treatment response, but with a reappearance of symptoms after the requisite decrease in medication dose. The latter was characterized by the emergence of a new reaction episode ≥3 months after discontinuing medical treatment, during which no reaction sign or symptom was observed [21].

Patients received monthly medication and went through medical, nursing, and physiotherapy consultations. After discharge from multidrug therapy, patients continued to attend the service every 3 months, except in cases where they experienced leprosy reactions. In those cases, patients were seen according to their needs, with shorter intervals between visits (15 days, on average). Patients were followed for a minimum of 2 years and a maximum of 5 years.

Clinical examination was initiated by inspection of all skin, recording the appearance, morphology, location, and number of lesions. Thermal, painful, and tactile sensitivity tests were subsequently performed. The patients also underwent additional testing, such as biopsies with histopathological and dermal lymph smears to facilitate the classification of the disease according to the Ridley and Jopling criteria. For treatment purposes, borderline tuberculoid patients were classified in the group of paucibacillary leprosy following negative smear results [22], with fewer than 5 lesions, and histopathological compatibility with this form.

The collected data were structured in a database using the Microsoft Excel 2007 program, which was also used to produce all tables and graphs representing data. The BioStat 5.0 software was used for statistical analysis, considering a confidence interval (CI) of 95%, and an α level of 5% (p-value ≤0.05). For quantitative variables, the measures of central tendency were used, followed by t-tests to compare quantitative variables between groups. Chi-square and G tests were used for comparisons of independent samples. To estimate and quantify the contribution of a variable for the occurrence of certain clinical results, the relative risk (RR) was used as the analysis of two variables’ measure of association. To analyze the occurrence of reactive states and estimate the risk of reaction, we used the Kaplan–Meyer test, which generated a survival curve over a period of 24 months.

### Ethics Statement

This research was approved by the Ethics Committee on Human Research at the Núcleo de Medicina Tropical da Universidade Federal do Pará under the protocol 001/2011.

Adult subjects had provided written informed consent in agreeing to participate in the study. For those participants who were under 18 years old, a written the informed consent was provided by their parents or guardians.

## Results

Of the 40 patients included in Group 1 and 107 patients in Group 2, 67.5% and 67.3%, respectively, were male; the predominant age group was 31–59 years for both groups, with the average age being 37 years (Table 1). The paucibacillary form accounted for 70% of cases found in Group 1, while the multibacillary form accounted for 80.4% of cases in Group 2 (chi-square, p < 0.0001). Patients without HIV infection were more likely to evolve into the multibacillary form of leprosy compared to co-infected patients (RR = 3.0) (Table 1). In Group 1, the predominant clinical presentation was borderline tuberculoid in 45% of cases, while in Group 2, the clinical type borderline was primarily expressed in 40.2% of cases (G test, p < 0.0001) (Table 1).

**Table 1:** Distribution of patients according to sex, age, and operational and clinical classification.

General characteristics	Studied groups				Statistical test
	HIV and Leprosy		Leprosy		
	N	%	N	%	
**Gender**					
Male	27	67.5	72	67.3	Chi-square
Female	13	32.5	35	32.7	p = 0.8623
Total	40	100	107	100	
**Age group (years)**					
≤15	1	2.5	12	11.2	G Test
16 to 30	10	25.0	31	29.0	p = 0.0872
31 to 59	28	70.0	55	51.4	
≥60	1	2.5	9	8.4	
Total	40	100	107	100	
**Age group (years)**					t-Test
Average ± Standard deviation	37.8 ± 10.4		36.2 ± 16.5		p = 0.4897
**Operational Classification**					
Paucibacillary	28	70	21	19.6	Relative Risk = 3.0
Multibacillary	12	30	86	80.4	p < 0.0001
Total	40	100	107	100	IC95% = 2.0–4.6
**Clinical Form**					
Pure Neural	0	0.0	3	2.8	
Indeterminate	3	7.5	4	3.7	G Test
Tuberculoid tuberculoid	7	17.5	14	13.1	p < 0.0001
Borderline tuberculoid	18	45.0	11	10.3	
Borderline borderline	10	25.0	43	40.2	
Borderline lepromatous	2	5.0	21	19.6	
Lepromatous lepromatous	0	0.0	11	10.3	
Total	40	100	107	100	

Source: Research Protocol, 2012.

Only 37.5% of patients in Group 1 had a leprosy episode, while 56.1% of patients in Group 2 did (chi-square, p = 0.0026). Comorbid patients were less likely to experience leprosy reactions (RR = 0.47) (Table 2). In both groups, the most frequent response was the Type 1 or reversal reaction, accounting for 86.7% and 56.6% of cases in Groups 1 and 2, respectively (G test, p = 0.0750). Acute neuritis was observed in 17.5% and 25.2% of patients in Groups 1 and 2, respectively, with no statistical difference between the two groups (Table 2). The treatment of the reaction manifestation was the same in both groups, with prednisone being the drug of choice for the reversal reaction, at a dose adjusted to 1 mg/kg/day; 14 and 36 patients from Groups 1 and 2, respectively, used this dosage of the drug. In the patients with a Type 2 response, thalidomide was the drug of choice (Table 2).

**Table 2:** Distribution of patients according to the clinical characteristics of leprosy reactions.

Clinical characteristics	Studied groups				Statistical test
	HIV and Leprosy		Leprosy		
	N	%	N	%	
**Leprosy reaction**					
Yes	15	37.5	60	56.1	Relative risk = 0.47
No	25	62.5	47	43.9	p = 0.0026
Total	40	100	107	100	IC95% = 0.28–0.79
**Type of reaction**					
Type 1	13	86.7	34	56.6	G test
Type 2	2	13.3	22	36.7	p = 0.0750
Type 1 and 2	0	0.0	4	6.7	
Total	15	100	60	100	
**Neuritis**					
Present	7	17.5	27	25.2	Chi-squared
Absent	33	82.5	80	74.8	p = 0.4414
Total	40	100	107	100	
**Prednisone dose**					
None	26	65	71	66.3	G test
40 to 50 mg	4	10.0	5	4.7	p = 0.6672
≥ 50 mg	10	25	31	29.0	
Total	40	100	107	100	

Source: Research Protocol, 2012.

Regarding the clinical form of the leprosy reactions, it was observed that, in comorbid patients, borderline tuberculoid was the predominant clinical presentation among patients experiencing the Type 1 reaction, at 61.6% of patients. Only 2 patients were observed to develop the Type 2 response; both belonged to the group presenting the borderline lepromatous manifestation. Patients without comorbid infections had both Type 1 and 2 reactions; the predominant clinical form in this cohort was the borderline borderline, with 57.9% of Type 1 patients and 38.5% of Type 2 patients displaying this form (Table 3).

**Table 3:** Distribution of patients correlating the clinical form with leprosy reaction type.

Clinical presentation	Leprosy reactions				Statistical test
	Type 1		Type 2		
	N	%	N	%	
**Leprosy/HIV**					
Tuberculoid tuberculoid	1	7.7	0	0.0	G test
Borderline tuberculoid	8	61.6	0	0.0	p = 1.00
Borderline borderline	4	30.7	0	0.0	
Borderline lepromatous	0	0.0	2	100.0	
Lepromatous lepromatous	0	0.0	0	0.0	
Total	13	100.0	2	100.0	
**Leprosy**					
Tuberculoid tuberculoid	1	2.6	0	0.0	G test
Borderline tuberculoid	2	5.3	0	0.0	p = 0.0638
Borderline borderline	22	57.9	10	38.5	
Borderline lepromatous	11	28.9	8	30.8	
Lepromatous lepromatous	2	5.3	8	30.8	
Total	38	100.0	26	100.0	

Source: Research Protocol, 2012.

The most prevalent type of reaction in both groups was the reversal reaction. The group of comorbid patients experienced skin lesions consistent with the expectations for each clinical presentation; the lesions were erythematous and infiltrated, with a similar progress and outcome as those found in patients without HIV, and responded appropriately to the use of prednisone. Generally, after 30 days of prednisone, dose-adjusted for weight, the patients' lesions had no infiltration and were in regression. Three of the 13 co-infected patients with reaction showed ulcerated lesions, but also had a good response to prednisone in the expected period of time. The same kind of ulcerated lesion in the Type 1 reaction was observed in 5 patients with leprosy alone.

The two coinfected patients experiencing Type 2 reactions showed the classic clinical manifestations, with widespread painful erythematous nodules on the body, as well as fever and arthralgia. Three of the patients without co-infection had ulcerated Type 2 reactions.

In those patients with HIV and leprosy experiencing a leprosy reaction, 14 (93.3%) were in the acquired immune deficiency syndrome (AIDS) state (chi-square, p = 0.0239), all of them on highly active antiretroviral therapy (G test, p = 0.0439). Another 7 patients (46.7%) had leprosy with a reversal reaction episode while experiencing immune reconstitution syndrome (G test, p = 0.0855) (Table 4). In these patients, we were able to quantify and observe a significant increase of serum T-helper CD4+ cells at the time of HIV diagnosis, prior to initiation of highly active antiretroviral therapy (average 141.8), compared to the time of diagnosis of the leprosy reactional state (average 367.7) (t-test, p = 0.0088).

**Table 4:** Distribution of patients exhibiting leprosy reactions with the characteristics of co-infected patients.

Coinfected characteristics	Leprosy reaction				Statistical test
	With reaction		Without reaction		
	N	%	N	%	
**Highly active antiretroviral therapy**					
Yes	15	100.0	18	72	G test
No	0	0.0	7	28	p = 0.0439
Total	15	100.0	25	100.0	
**AIDS**					
Yes	14	93.3	14	56	G test
No	1	6.7	11	44	p = 0.0239
Total	15	100	25	100.0	
**Immune reconstitution inflammatory syndrome**					
Yes	7	46.7	4	16	G test
No	8	53.3	21	84	p = 0.0855
Total	15	100	25	100	

Source: Research protocol, 2012.

In the first 6 months of observation, there were more leprosy reactions in both groups. At the end of these sixth months, 67.5% of Group 1 patients did not have any kind of leprosy reaction, neither did 74.77% of Group 2 patients. After the 24 months of observation, both groups behaved similarly and remained stable; 65% and 63.55% of patients in Groups 1 and 2, respectively, had had no reaction during this time, with no new patients experiencing reactions as of 18 months since the beginning of multidrug therapy.

Most patients (73.3% in Group 1 and 75% in Group 2) experienced only one cycle of leprosy reaction. However, no patient in Group 1 had more than three episodes, while 10 (16.7%) patients in Group 2 did (G test, p = 0.0371) (Table 5). Further, 93.3% of patients in Group 1 had relatively short leprosy reaction cycles, of ≤3 months, while 63.3% of patients in Group 2 experienced longer cycles of over 3 months' duration (p >0.0001). Patients without co-infection were more likely to have reactional states of over 3 months compared to the comorbid patients (RR = 7.5) (Table 5).

**Table 5:** Distribution of patients according to clinical characteristics during the reactional states.

Clinical Characteristics	Studied Groups				Statistical test
	HIV and Leprosy		Leprosy		
	N	%	N	%	
**Number of reactional cycles**					
1	11	73.3	45	75.0	G test
2	4	26.7	5	8.3	p = 0.0371
≥3	0	0.0	10	16.7	
Total	15	100	60	100	
**Cycle duration (months)**					
≤3 months	14	93.3	22	36.7	Relative risk = 7.5
>3 months	1	6.6	38	63.3	p <0.0001
Total	15	100	60	100	CI95% = 2.4–23.4
**Severity of reaction**					
Mild	0	0.0	3	6.3	G test
Moderate	12	80.0	26	54.2	p = 0.1577
Severe	3	20.0	19	39.6	
Total	15	100	48	100	
**Type of repetition**					
Subintrant	1	20.0	15	41.7	G test
Recurrent	4	80.0	21	58.3	p = 0.6540
Total	5	100	36	100	

Source: Research Protocol, 2012.

As for reaction severity, most of the patients in both groups showed episodes of moderate severity: 80% in Group 1 and 54.2% in Group 2 (G test, p = 0.1577) (Table 5). Most of the patients in the two groups also had recurrent episodes, representing 80% and 58.3% of patients in Groups 1 and 2, respectively (G test, p = 0.6540) (Table 5).

## Discussion

This paper outlines a clinical follow-up performed on a group of patients infected with *M*. *leprae* and HIV (co-infected; Group 1) and another group of patients infected with *M*. *leprae* alone (Group 2). Important characteristics of these patients were observed, emphasizing their clinical aspects, and the occurrence and characteristics of leprosy reactions. In general, clinical and epidemiological studies of leprosy with or without comorbidity show no significant predilection towards patients’ gender, but most of the cases involved male patients. For example, in the research of Lima, Prata, and Moreira [23], 1,940 cases of leprosy were found in men, and men have also predominantly been affected in other studies as well [24–26]. In co-infected patients, men have been the majority of those affected in three of the four largest existing groups studied to-date [8,17,27].

The adult age group of 31–59 years was the most represented in both groups in this study (Group 1: 70%, Group 2: 51.4%), consistent with most other studies. The increased risk of being affected by leprosy in adult life has been associated with the increased exposure of this highly economically-active age group [8,23,24,27].

Although an increasing number of cases of multibacillary leprosy was predicted at the beginning of the HIV/AIDS pandemic [11,28], the present study shows that HIV infection tended to associate with paucibacillary leprosy (70%). On the other hand, the group with leprosy alone had significantly higher cases (80.4%) of multibacillary leprosy (p <0.0001).

The predominance of paucibacillary leprosy in the co-infected group is consistent with many previously published studies on this type of comorbidity [7,8,10,11,15–17,29–34]. The early diagnosis of these patients, while they are at the paucibacillary clinical form, is recommended. During this phase, lesions are still not clinically apparent in most cases, and patients may experience their clinical condition, following a diagnosis during the course of immune reconstitution inflammatory syndrome after the beginning of antiretroviral therapy. It can be speculated that most of the patients experiencing a comorbidity of HIV and leprosy would not manifest leprosy if they were not infected with HIV. It has been suggested that, among HIV-infected patients, the diagnosis of leprosy has been associated with patients’ immune improvement, characterized by elevated T-helper CD4+ lymphocyte counts and lower viral loads. Therefore, the appearance of clinical signs of *M*. *leprae* infection in HIV patients would, in fact, not be a manifestation of immune suppression, but the immune reconstitution that follows the occurrence of highly active antiretroviral therapy [11,17,35,36].

The predominance of multibacillary leprosy in Group 2 is also consistent with several studies originating from leprosy epidemiology services [24,25,28,37,38]. This is possibly due to their specialized services, addressing more complex cases of leprosy that are more difficult to manage clinically, such as multibacillary leprosy.

Using the classification of Ridley and Jopling for both groups, we found that the prevailing clinical form in Group 1 was borderline tuberculoid (45%). The largest series of cases of HIV comorbid with leprosy were studied in Brazil, which showed that the majority of cases were manifestations of the borderline tuberculoid form, including isolated cases where this clinical manifestation is also prevalent [8,17,18,25,32,37]. The published study observing the largest number of cases on this subject, performed by Mehta et al [17], followed 100 patients from 1989 to 2010, and found that the majority of cases of paucibacillary leprosy could be classified as the borderline tuberculoid clinical form. In another representative study on this co-infection, Xavier [34] studied 31 patients, of which 49% were classified as borderline tuberculoid. In Group 2, there was a predominance of borderline borderline (40.2%) and borderline lepromatous (19.6%) cases, consistent with what has been shown in other studies in Minas Gerais and Mato Grosso do Sul [28,39].

In this clinical cohort of co-infected patients, no atypical clinical manifestations or "changes" of clinical forms were observed, similar to previous reports [35]. Although it was expected that the clinical forms or clinical course of patients with leprosy and HIV/AIDS would be more serious, as has been observed in other cases of comorbidity, worsening or different manifestations have not yet been found for leprosy [8,9,17,38–41]. Some cases have been described involving ulcerative lesions as a different clinical manifestation, but it was later concluded that these cases were, in fact, a reversal reaction, in which ulceration can occur due to severe swelling in the dermis [42], even in patients without co-infection [7,14,16]. Studies have reported a case initially believed to be a transformation from borderline tuberculoid to the lepromatous lepromatous clinical form, called down grading [10]. In this case, down grading was described as having occurred when the patient was not receiving multidrug therapy, indicating a natural evolution of the disease. However, more in-depth analysis of the patient's clinical history indicated that the first treatment had not been finalized and, subsequently, the clinical manifestation worsened due to treatment failure, not HIV co-infection [10]. On the other hand, a study of 25 co-infected patients described the contrary, reporting that 2 patients with the borderline lepromatous clinical form exhibited a "shift" of "improvement" to the borderline tuberculoid form, which the authors described as a possible "upgrading" after a Type 1 reaction, even with the patients being HIV-positive [18].

During the clinical follow-up of patients for a period of at least 2 years, the critical period for the occurrence of leprosy reactions [43–45], only 15 patients (37.5%) of the co-infected group experienced some type of leprosy reaction, while in the non-comorbid group, 60 patients (56.1%) presented these reactions. Within this significant difference in leprosy reactions, we calculated that the co-infected patients had a lower chance of developing reactive frameworks compared to the non-comorbid group (RR = 0.47; p = 0.0026). In both groups, the reversal reaction was predominant, occurring in 13 (86.7%) and 34 (56.6%) patients in Groups 1 and 2, respectively.

The largest occurrence of reversal reaction in leprosy patients without HIV is found when there is a predominance of borderline clinical forms, since these are immunologically unstable [24,26,39,45–47]. The fact that the group of non-comorbid patients also had a significant number of borderline lepromatous explains the significant occurrence of Type 2 reactions, which primarily occur in patients with this clinical form, in 22 patients (36.7%). In Group 1, only 2 patients (5%) experienced this kind of reaction, but these 2 patients exhibited the borderline lepromatous clinical form. The cases that have been previously reported, in which a co-infected patient presents a Type 2 reaction, occurred in patients who were already severely immunosuppressed for a prolonged period of time, contrary to the situation of the patients observed in the present study [48,49].

In Group 1, 8 of the 13 patients (61.6%) exhibiting a reversal reaction had a borderline tuberculoid clinical form; of these 8 patients, 6 manifested immune reconstitution inflammatory syndrome, as described in several other studies [8,16–18,35,40]. Co-infected patients had reactional lesions, both Types 1 and 2, with aspect, and an expected clinical evolution similar to patients in Group 2.

The treatment of these reactional states, including acute neuritis in the co-infected group, was performed using the same prescribed medication to patients with leprosy only, following the normative of the latest ministerial decree of 2010. This decree highlighted a preference for prednisone, at a dose of 1 mg/kg for Type 1 reactions, acute neuritis, and some cases of Type 2 reactions, and recommended the use of thalidomide for Type 2 reactions, with the exception of treating women of childbearing age [47,49]. Several published clinical studies also refer to the classical treatment of reactional states in co-infected patients, including an emphasis on the introduction of an appropriate dose of prednisone early to avoid scarring, especially in cases of acute neuritis and concomitant immune reconstitution inflammatory syndrome [17,18,31,41,50]. The majority of patients in Groups 1 and 2 (25% and 29%, respectively) received over 50 mg of prednisone in accordance with their weight, with a subsequent gradual reduction according to clinical improvement. In most cases, the reduction occurred every 15–20 days, decreasing to a dose of 20 mg, then 5 mg, and finally a complete withdrawal.

All of the 15 co-infected patients with a leprosy reaction were using highly active antiretroviral therapy (p = 0.0439) and 14 (93.3%; p = 0.0239) were in the AIDS stage and still developing a reactive state. This has also been described in other studies [17,35], demonstrating that several factors may influence the immune behavior of both diseases. Of the 13 co-infected patients exhibiting a reversal reaction, 7 (53.85%) also manifested leprosy as a result of immune reconstitution inflammatory syndrome within the first 6 months of using antiretroviral therapy, according to the criteria described by Deps and Lockwood [6] and used in other studies of co-infection [17]. However, there have been cases where patients who already have leprosy, including those in specific treatment with multidrug therapy, only presented reactions after initiation of highly active antiretroviral therapy [51].

There have been several reports previously published that are consistent with our data regarding patients without a comorbidity. Particularly, many report that the first reaction episode usually occurs during treatment with multidrug therapy or even 6–12 months after the end of the multidrug therapy [1,44,50].

Due to the lack of studies examining co-infected patients, there are very few details regarding the timing, duration, and number of cycles of reactional states; the literature generally discusses only the reversal reaction as a manifestation of immune reconstitution inflammatory syndrome [8,16–18,34,40]. For these patients, the possible, potentially unregulated, immunological improvement is the underlying cause of the cellular immune response against the *M*. *leprae* antigen before the start of multidrug therapy, even if the bacilli are still intact, which may explain the high incidence of leprosy reversal reaction cases [31].

The present study describes in more detail the number, timing, and degree of reactional state cycles in leprosy patients with HIV comorbidity. In both groups, most of the patients had a single episode of leprosy reaction within 2 years of follow up (73.3% in Group 1, 75% in Group 2). Both groups also showed reactive episodes of moderate intensity (80% in Group 1, 54.2% in Group 2), with a larger number of more serious conditions in Group 2 patients (39.6%), all of which is consistent with previous reports on the subject [25,29,38,45,50]. We observed that the group of patients with leprosy alone was more likely (RR = 7.5) to have extended reactional states, lasting for more than 3 months, while almost all co-infected patients (93.3%) had short periods of leprosy reaction, averaging at 2 months.

This significant difference in the duration of reactive episodes between groups may be related to several factors, including distinct immunological states, since most of the co-infected patients experiencing reversal reactions also manifested AIDS, entering immune reconstitution inflammatory syndrome (93.3%). However, one of the associating factors that cannot be overlooked is that patients in Group 2 presented the highest number of multibacillary leprosy cases (borderline and borderline lepromatous), which are more immunologically unstable. It was also proven that the more bacilliferous the patient, the greater is the chance of having longer-lasting reactive episodes [46]. Further, patients with a bacterial index ≥2 have a higher risk of reactional episodes, including a greater chance of subintrant episodes, as seen in 41.7% of cases this study. These would be cases in which outbreaks occur so frequently that they appear continuous, which may explain why 63.3% of Group 2 patients experienced longer episodes [45,46]. This study did not observe the patients' bacterial indices, since the validity of this calculation depends on well-trained laboratory technicians and constant quality control in order to reduce the risk of errors.

### Conclusion

The follow-up study of two clinical cohorts of leprosy patients, one experiencing comorbidity with HIV/AIDS and the other not, found that the observed dermatological lesions had a usual aspect with no significant difference between groups, and good clinical progress with the administration of prednisone, the preferred therapeutic for leprosy reaction. There was no significant difference in the prevalence of leprosy reactions between the two groups, and both groups predominantly experienced a Type 1 response, with only one reaction event. Co-infected patients exhibited moderate reaction severity, with predominantly shorter cycles. Although many questions remain in the study of leprosy and HIV comorbidity, particularly regarding leprosy reactions, this work provides information able to confirm assertions that such diseases, when concurrent, are independent in their progression. Future studies may wish to further examine the relationships between the two diseases to corroborate these conclusions.

## Supporting Information

S1 Checklist STROBE checklist for cohort studies. (DOC)
